# Direct and Indirect Effects of Belief in a Just World and Supervisor Support on Burnout via Bullying

**DOI:** 10.3390/ijerph15112330

**Published:** 2018-10-23

**Authors:** Pascale Desrumaux, Nicolas Gillet, Caroline Nicolas

**Affiliations:** 1 Department of Psychology, Laboratory Psitec, University of Lille, 59650 Villeneuve d’Ascq, France; caroline_306@hotmail.com; 2 Department of Psychology, Laboratory PAV, University of Tours, 37020 Tours, France; nicolas.gillet@univ-tours.fr

**Keywords:** workplace bullying, burnout, supervisor support, belief in a just world, mediation

## Abstract

The aim of the study was to examine the mediating role of workplace bullying in how supervisor support and belief in a just world (BJW) are related to emotional exhaustion. A cross-sectional quantitative study using anonymous self-report questionnaires was conducted with 434 workers in France. The model was tested using a path analysis. First, the results revealed that BJW and support from the hierarchy were negatively related to emotional exhaustion. BJW and supervisor support were also negatively related to workplace bullying. Finally, harassment at work was positively related to emotional exhaustion. More generally, the results showed that social support from one’s superior and BJW were directly and indirectly related to emotional exhaustion via bullying at work. Our model thus suggests that BJW and supervisor support can be protective resources against bullying and exhaustion. We discuss the theoretical and practical implications of the present study, as well as some avenues for future research.

## 1. Introduction

Burnout, an international health problem that has heavy spinoffs for organizations, persons, and societies, is defined as an affective reaction to ongoing stress, whose main dimension is emotional [1] and is rooted in the depletion of emotional resources. The causes of burnout are generally divided into two categories: situational factors and individual factors [2]. Situational factors include job demands and lack of job resources such as a lack of support. In contrast, psychological, social, and organizational job resources can protect a person from burnout by facilitating the achievement of work goals and reducing job demands and overload [3]. In explaining and studying burnout, researchers have generally focused on either organizational factors (e.g., job demands or job resources) or individual factors (e.g., self-efficacy, self-esteem, and self-determination). In doing so, they have ignored the role of psychosocial factors such as work relations and human beliefs about burnout. Few studies have investigated the role of serious conflicts that degenerate into psycho-terror or bullying [4,5]. An analysis of the 2010 US National Health Interview Survey data [6] showed for a sample of 17,524 adults that 8.1% reported being harassed at their workplace. Harassment was associated with psychosocial distress, pain disorders, work loss, sick days, and worsening health among employees.

An overview of the literature shows that bullying and burnout have rarely been simultaneously investigated in an attempt to understand the joint roles of causal factors such as beliefs and social support. A meta-analysis of 25 studies [7] performed in Europe, North America, Australia, and New Zealand indicated that a heavy workload, few rewards, little supervisor support, and low co-worker support were associated with emotional exhaustion. Yet, workplace bullying and burnout are linked, for several reasons. First, they share common causes such as job characteristics (e.g., high demands and a low amount of resources), lack of social support, abusive management, bad work climate, and unfairness. In particular, the absence of social support can increase bullying [8] and burnout [9]. Second, bullying is linked to burnout because repeated stressful conflicts and situations [10] or a power imbalance between the parties [11], which are the well-known characteristics of bullying, will necessarily lead to exhaustion. Third, high emotional demands generated by conflicting situations or by attacks upon a person (e.g., verbal attacks, humiliations, and physical attacks) will produce emotional strain and emotional wear, and can account for the loss of emotional resources and physical and emotional collapse [12].

Based on a social exchange perspective, our study looks into the direct and indirect roles of social support and belief in a just world (BJW) in explaining burnout, and more specifically emotional exhaustion. BJW can act as a positive coping mechanism with many psychological benefits [13]. In fact, BJW can play two complementary adaptive functions: in the prevention view, it helps individuals reduce negative psychological symptoms, and in the promotion view, it helps individuals achieve a positive psychological state [14]. Why can we consider BJW and support to be protective factors in the face of burnout? In this paper, we see BJW and support as resources. Resources can offset the negative effects of conflicts on mental health and specifically on burnout [15]. When valued resources like supervisor support are lost, individuals are likely to experience negative consequences that include emotional exhaustion [16].

Firstly, support from supervisors and colleagues can alleviate burnout because it provides employees with important relational (e.g., cooperation and energy), emotional (e.g., positive affect and positive recognition), informational (e.g., training, counseling, and demonstration of practices), and tangible resources (e.g., sharing of demands). Some authors [17] have found that, when employees had social support, or had a high-quality relationship with their supervisor, being subject to work overload and emotional demands did not result in high levels of burnout.

Secondly, beliefs such as the belief in equity or the belief in a just world can reduce uncertainty and anxiety, especially in stressful and conflicting situations. The literature on BJW has provided evidence of two views (prevention/promotion) that demonstrate the adaptive functions of BJW [14]. According to the first view, BJW helps individuals reduce negative psychological symptoms [18]. Studies showed, for example, that strong believers report less emotional exhaustion and fewer symptoms of depersonalization [19]. Transferred to the workplace, BJW not only provides a conceptual framework that helps individuals to interpret uncertain, uncontrollable (e.g., one’s workload) or difficult job events in meaningful ways but also enables people to place more trust in other employees, believe that they will be treated fairly by others (supervisor and colleagues), and feel that they will not be victims of uncertainty, conflicts, personal attacks, or problems. According the promotion view, BJW helps individuals promote a positive psychological state [20].

More specifically, reinforcing prevention requires exploring the direct and indirect roles of occupational support and BJW. This study was aimed at understanding how a combination of supervisor support and BJW affects perceived bullying, which in turn can lead to emotional exhaustion.

### 1.1. Perceived Social Support, Psychological Health, and Burnout

Many research studies have demonstrated the beneficial effects on burnout of co-worker support and supervisor support [21]. Both the main effects and buffering effects of support have been demonstrated in the burnout literature [21,22]. For example, the authors of [22] found that informational and instrumental support provided by both co-workers and supervisors had buffering effects on burnout in social workers. However, two questions need to be explored. First, due to the paucity of research on the role of social support in burnout when workers are bullied, it is important to find out whether social support mediates the effects of exposure to job suffering. Furthermore, if workers are exposed to bullying, what roles can supervisor support play? Second, the role of BJW as a protective belief [23] from bullying and burnout has not been explored. Moreover, although relationships between bullying and outcomes (e.g., burnout) have been established, little is known about the psychological mechanisms (i.e., when bullying acts as a mediator) that lead to job exhaustion.

It is necessary to focus on the relationships between health (burnout and bullying), social support from one’s supervisor, and beliefs for at least two reasons. First, social support is known to be related to these two psychosocial predictors (burnout and bullying). Social support is therefore an inescapable and major basis. Second, to our knowledge, the relationship between burnout and bullying has never been explored in relation to BJW. The first objective of this study is to gain insight into how supervisor support can protect workers from these two major risks, burnout and bulling. Our second objective is to understand the mediating role of bullying in the relationship between BJW and burnout.

### 1.2. Perceived Supervisor Support, Burnout, and Emotional Exhaustion

The concept of social support is rooted in the workplace in social exchange theory [24]. Employees engage in various exchanges within the organization with their immediate supervisor [25], and with colleagues. Support and favorable treatment by supervisors enhances employees’ perception that the organization (including management) values their contributions and cares about their health and well-being [26]. Thus, perceived supervisor support is partially related to organizational support [27]. From the supervisor–employee angle, in order to achieve a balance, each party must offer something that the other party considers valuable, and each party must perceive the exchange as fair and just. Scholars [28] who distinguish supervisor and colleague support consider that supervisor support can include material and technical support, receptivity support (listen and pay attention to employees), initiative support (autonomy left to employees), and authority support (respect for the rules).

Social support plays an important role in reducing chronic stress at work and in reducing burnout as well [29,30]. The links between a lack of support and burnout are well known. Negative relationships with co-workers and supervisors have been shown to aggravate burnout. Social support appears to be associated with every dimension of burnout [9].

Perceived social support is related to a low level of burnout among teachers [21] and nurses [9,31], and their workplace bullying and emotional exhaustion can be reduced through the social support they receive from people around them [32]. Lack of support from one’s supervisor is a source of professional stress, and to a large extent, engenders the risk of professional exhaustion [33]. The lack of supervisor and colleague support is significantly related to the three dimensions of burnout. Moreover, verbally abusive supervision has been empirically linked to burnout [34].

Studies have shown that the greater the number of social-support channels, the greater the capacity of individuals to cope with emotional exhaustion [32]. Emotional exhaustion is characterized by feelings of exhaustion, a lack of energy, and physical and emotional overload. Emotional exhaustion is considered the key factor of burnout and appears when there is an imbalance between expectations related to one’s work life (in terms of, e.g., fair management and regular workload) and the reality of daily work (in terms of lack of supervisor support, pressure, etc.). For this reason, many studies focus solely on this major dimension of exhaustion, and there are several scales that measure only the attitudinal component of the burnout syndrome, in which only exhaustion plays a prominent role [35], while failing measure depersonalization and reduced personal accomplishment. Many studies have found specific effects of emotional exhaustion [36].

In the scientific literature, these two secondary dimensions (depersonalization and reduced personal accomplishment) have been modified and reduced to cynical attitudes and a lack of self-efficacy. Some authors have suggested that reduced efficacy need not be measured as a part of burnout [33]. On the contrary, for scientific and methodological reasons, we prefer to keep exhaustion. As a result of ongoing interactions between workers and job demands, emotional exhaustion is slowed down by social support. Researchers [21] have reported that practical, emotional, and informational support from one’s supervisor is negatively related to emotional exhaustion. Studies have also shown that perceived social support from superiors, as a protective factor, is negatively related to burnout among managers [37].

Based on several studies wherein a lack of supervisor support predicted exhaustion [21,22,37], our first hypothesis is as follows:

**Hypothesis** **1.** *Supervisor support is negatively related to emotional exhaustion.*

### 1.3. Perceived Social and Supervisor Support, and Workplace Bullying

Workplace bullying has an impact on mental and physical health [38] and leads to extreme feelings (learned helplessness or distress) or extreme self-directed behaviors such as suicide [39,40]. Mobbing was defined [41] as a sequence of actions that occurs frequently (at least once a week) over a rather long period, and consists of comments and hostile schemes expressed by one or more persons toward a targeted individual. At the workplace, it consists of repeated and prolonged infringements of an employee’s personal dignity. Many studies [10,11,42] have shown that both personal and organizational factors contribute to determining the likelihood of bullying.

According to many scholarly studies [11], it has been shown [41] that bullying is primarily caused by factors related to poor organization at the workplace and deficient leadership behavior within the organization. In a recent Finnish study, leadership and job demands were significantly associated with bullying. Constructive leadership was associated with lower levels of bullying, while higher reported levels of job demands were associated with a risk of bullying that was four times that of those with lower reported job demands [43].

Such work-environment characteristics may provoke bullying directly, but they may also contribute to creating a stressful work climate in which bullying can flourish [5,44].

Many studies have underlined the lack of social support from superiors and coworkers toward a bullied target [11]. While a few people may support the target, others may not offer any help at all, and sometimes even turn against the target [41]. It is often observed that superiors ignore the situation and do not intervene, in such a way that a lack of social support has an impact on bullying and emotional exhaustion [8]. Recent research has tried to explain bullying by taking into account others’ judgments of the perpetrators, the victim, and the bullying situation. A few studies have shown that personal and organizational characteristics (e.g., climate and social norms) influence judgments of fairness and responsibility [45]. Thus, bullying acts are judged fairer when the climate is authoritarian and based on strict rules and procedures. In these climates, supervisors rarely help subordinates and try to control them. In contrast, climates that favor autonomy and supervisor support decrease judgments of fairness in bullying situations. In a study [46], bullying was correlated with bad job content, a poor social environment, and psychological ill health. The findings suggested that the more social support supervisors gave, the less the victims reported being shouted at, being constantly criticized, and receiving verbal threats. These studies led us to set forth the following hypothesis:

**Hypothesis** **2.** *Perceived supervisor support is negatively related to workplace bullying.*

### 1.4. Bullying and Burnout

A few studies have considered burnout to be a potential negative consequence of bullying [47,48,49]. In fact, burnout, and more specifically emotional exhaustion, have been consistently linked to exposure to workplace bullying [47]. In health care services, for example, authors have shown [47], with a sample of 2700 nurses, that bullied nurses had significantly higher levels of burnout. Other scholars [50] also found that bullying was positively associated with burnout in nurses.

One reason why bullying can affect burnout has to do with the strain that results from bullying acts. However, many authors support the hypothesize that chronic strain is an antecedent of burnout. Given the frequency, persistence, diversity, and synergy of acts of bullying, it can be experienced not only as an extreme source of social stress at work [10] but as a continuous source of strain, thereby leading to exhaustion. A second reason why bullying can induce burnout is that bullying situations decrease satisfaction, pride, and strength, induce a lack of autonomy, and increase the thwarting of psychological needs that are sources of energy, vitality, and morale. In this vein, a study [51] among 1179 nurses in Quebec supported a model that included satisfaction needs as mediators. They found that workplace bullying positively predicted burnout, via a lack of satisfaction of employees’ need for autonomy. Later studies on nurses confirmed that bullying increases burnout symptoms [48,52]. The results [52] showed that workplace bullying thwarted the satisfaction of employees’ basic psychological needs and fostered burnout 12 months later. A third reason why bullying affects burnout is linked to emotions. Emotional abuse, a frequently used term to characterize bullying and to capture the hostile verbal and nonverbal behaviors that injure employees morally, is likely to affect the person’s emotional balance and deeply affect his/her potential resources because it deprives this person of satisfaction, energy, and freedom of thought.

Consequently, in line with many studies linking workplace bullying to burnout [38,49,51,53], the present study contends that workplace bullying predicts emotional exhaustion. Our third hypothesis is as follows:

**Hypothesis** **3.** *Exposure to workplace bullying is positively related to emotional exhaustion.*

The role of bullying has been investigated as a mediator between organizational factors and illness. For example, scholars [54] have found that mobbing mediated the link between the lack of organizational support (organizational silence) and the intention to quit. In the same vein, the effects of laissez-faire leadership on distress appear to be mediated through exposure to bullying [55]. Additionally, negative emotions experienced in the case of emotional abuse and negative acts can mediate the relationship between support and emotional exhaustion. Using a field sample of 262 employees working in different organizations, scholars [8] have found that workplace bullying was a mediator of the relationship between perceived organization politics and perceived organization support, and emotional exhaustion. The social support of supervisors may protect workers from workplace bullying [56] and emotional exhaustion [57]. We also hypothesize here that the lack of supervisor support is conducive to the development of workplace bullying, which in turn promotes emotional exhaustion. Our fourth hypothesis is as follows:

**Hypothesis** **4.** *The effect of perceived supervisor support on emotional exhaustion is mediated by workplace bullying.*

### 1.5. Belief in a Just World, Bullying, and Burnout

According to the just-world hypothesis, “people want to and have to believe they live in a just world so that they can go about their daily lives with a sense of trust, hope, and confidence in their future” [58]. As a resource, belief in a just world can have compensatory effects for dealing with stress at the workplace [19,59,60]. Believing the world is just, a place where one gets what one deserves, and deserves what one gets, is an important personal resource that helps maintain well-being at work. According to the belief in a generally just world, everyone is treated justly [20,61]. A study showed that the justice motive could act as a personal resource for dealing with challenges and critical life events [59].

As in many models on occupational health based on the balance concept (such as the effort-reward model [62], the job demands–resources model [3], and the conservation of resources theory (COR) [15]), it is possible that BJW works as a resource in the principle of a balanced representation of the world. In line with the demands–resources balance, or the balance between loss or threat of loss and assets in the conservation of resources theory, beliefs in a just world can function as a resource. COR theory extends prior theories by acknowledging that stress (and possible subsequent exhaustion) stems from the combined effect of the subjective perception of an event as taxing or exceeding available resources, and the objective environmental circumstances that threaten or cause depletion of resources. On that basis, BJW involves a subjective perception of events. Believing that the occupational world allows one to find fairness in everyday relations suggests that employees are treated with justice and fairness (in procedural, distributive, and informational manners), and this can unburden employees and mitigate the feeling of being worn out, overlooked, or abused at work.

Several authors [20] support the idea that general and personal beliefs in a just world need to be distinguished, because the personal BJW reflects the belief that, overall, the events in one’s life are just, whereas the general BJW reflects the belief that the world is basically a just place. Based on a general overview of the experiments conducted between 1980 and 2005, none of the studies in the review of 66 studies on BJW [63], looked at the relationship between BJW and bullying, but a few recent studies have shown relationships between BJW, distress, and bullying.

Testing the hypotheses that the personal BJW is correlated with school distress, some authors [64] found that BJW can best be interpreted as a personal resource (main effect) than as a buffer (moderator) for the distress of victims, bullies, and defenders of victims. More specifically, among boys, bullies experienced more distress and defenders experienced less distress at school. Personal BJW has been found to be less sensitive to negative life experiences than general BJW [61]. In this vein, one study [65] suggested that personal BJW exerts its influence on well-being by increasing the overall justice perceptions of the work environment. Consequently, personal BJW can be seen as a form of resilience that helps individuals diminish stress and distress despite significant adversity [66]. In particular, personal BJW was found to be negatively related to emotional exhaustion [19,60]. The theoretical and empirical considerations raised above led us to hypothesize the following:

**Hypothesis** **5.** *Belief in a just world is negatively related to emotional exhaustion.*

Another study [67] showed that BJW and emotional responses to negative outcomes was mediated by attributions: strong believers in a just world made stronger internal and weaker external attributions for their negative outcomes, leading to reduced perceived unfairness, which in turn led to less negative and more positive emotions.

However, this positive function of BJW may be altered because repeated and prolonged exposure to mobbing has adverse effects on BJW [68]. The results of these studies showed that perceived victimization by mobbing (rather than the mere frequency of exposure to negative acts at the workplace) was associated with worse adjustment and a weaker belief in the justness of the world. For some scholars [68], the belief in an unjust world is associated with a more negative, cynical, and pessimistic outlook on the world, and aggravates rather than alleviates the distress caused by perceived misfortune [66]. In another study [69], the feeling of being treated unfairly led to major job stress, which in turn led to exhaustion.

In fact, we can expect that being confronted with personal evidence that the world is not just and that one has no control over personal or occupational events, especially in cases of mobbing, increases the degree to which depression and exhaustion are experienced following the exposure to mobbing. These relationships between BJW, mobbing, and exhaustion bring us our final hypothesis:

**Hypothesis** **6.** *The effect of belief in a just world on emotional exhaustion is mediated by workplace bullying.*

## 2. Method

### 2.1. Participants

The questionnaire was filled in by 434 workers from two regions of France (Hauts-de-France and Pays-de-la-Loire). The sample included 176 males (40.6%) and 258 females (59.4%) age 18–65 (*M* = 38.25, *SD* = 11.25). A large majority of the participants worked during the day (95%) (5% did not answer this question). Most (87.8%) had worked for more than 10 years in the company and 12.2% had less than 10 years of seniority. Twenty-three percent were living alone and 77% were living with a partner; 48.8% had no dependent children and 51.2% had at least one dependent child. Participants were working in 70 companies: 28% in the public sector, 70% in the private sector, and 1% in the para-public sector. The most frequent sectors were commercial (21%), educative (12%), medical (8%), and industrial (8%). The questionnaires (paper version), given directly to employees or sent by postal mail (*N* = 377), were returned to members of the research team by mail or in person. Other respondents (*N* = 57) responded to the survey on line. Analyses were conducted in order to examine the possible effects of the data collection mode on the variables studied. No differences in the means were found for any of the variables.

### 2.2. Materials and Procedure

All questionnaires were administered in French. The questionnaire was presented as a survey on interpersonal relationships at work.

Personal belief in a just world [70] was measured using the 3-item validated French version of Personal Belief in a Just World Scale, which for practical reasons was made shorter than the initial English version by authors [20]. The items were rated from 1 (strongly disagree) to 6 (strongly agree). The scale assessed the belief that events in one’s life are just (e.g., “I am usually treated fairly”). Scores were computed by averaging across items, with higher scores indicating a stronger Personal BJW. Cronbach’s alpha was 0.78.

Social support from the supervisor (e.g., “My boss facilitates work”) was measured using an 11-item scale [71]. The participants ranked the extent of their agreement or disagreement on a 4-point Likert scale ranging from 1 (“I strongly disagree”) to 4 (“I strongly agree”). Cronbach’s alpha was 0.78.

Workplace bullying was determined using the Belgian version of the Negative Acts Questionnaire or NAQ [72]. The 16 items on this questionnaire measure the frequency of exposure to negative behaviors considered to constitute bullying at work if they occur regularly. The items are formulated in behavioral terms, with no reference to the expression “bullying” (e.g., “Spreading gossip and rumors about you”). On a four-point scale ranging from 1 (never) to 5 (daily), the participants were asked to indicate how frequently they had experienced each behavior within the last six months. The psychometric properties of the French version of the NAQ-R are similar to those of the original [73]. Cronbach’s alpha was 0.93.

Emotional exhaustion was assessed using the Maslach Burnout Inventory-General Survey (MBI-GS) [74]. Emotional exhaustion (e.g., “I feel used up at the end of a work day”) was assessed on five items. All items were rated on a seven-point scale ranging from 1 (never) to 7 (every day). Cronbach’s alpha was 0.89.

## 3. Results

### 3.1. Preliminary Analyses

The means, standard deviations, and latent correlations of the measures are presented in Table 1. An examination of the size and direction of the correlations (see Table 1) provided good preliminary support for the hypotheses. Perceived supervisor support (*r* = −0.56, *p* < 0.001) and belief in a just world (*r* = −0.40, *p* < 0.001) were negatively correlated with bullying. Moreover, perceived supervisor support (*r* = −0.43, *p* < 0.001), belief in a just world (*r* = −0.42, *p* < 0.001), and bullying (*r* = 0.59, *p* < 0.001) were significantly correlated with emotional exhaustion.

### 3.2. Main Analyses

**Figure 1:**
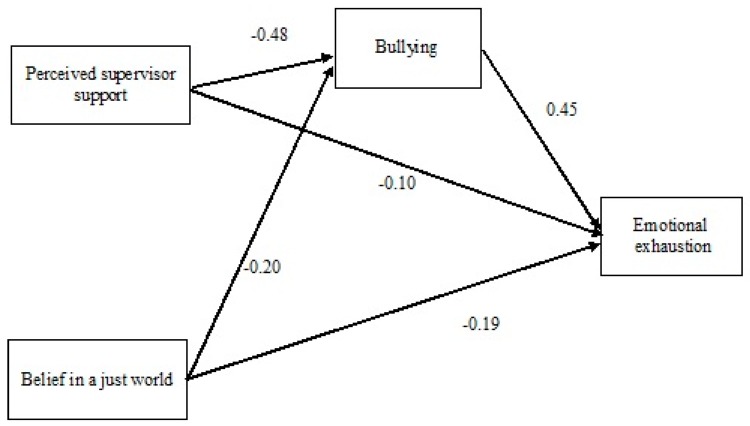
Results of the path analysis. Note: Standardized coefficients are reported. All paths are significant (*p* < 0.05).

As hypothesized, bullying was defined as a partial mediator in the relationship between perceived supervisor support and emotional exhaustion and between belief in a just world and emotional exhaustion. The hypothesized model was tested via a path analysis using the AMOS software. The analysis was conducted on the covariance matrix, and the solutions were generated on the basis of maximum likelihood estimation. Our hypothesized model was fully saturated, so we do not provide fit indices (see Figure 1). Perceived supervisor support (β = −0.48, *p* < 0.001) and belief in a just world (β = −0.20, *p* < 0.001) were negatively linked to bullying. Furthermore, perceived supervisor support (β = −0.10, *p* < 0.05), belief in a just world (β = −0.19, *p* < 0.001), and bullying (β = 0.45, *p* < 0.001) were significantly associated with emotional exhaustion.

To determine whether bullying acted as a mediator between perceived supervisor support and emotional exhaustion and between belief in a just world and emotional exhaustion, a bootstrapping approach was used [75]. Bootstrapping is a method that uses a given number (1000 in this study) of resamplings of the original sample and provides confidence intervals (CIs) for the estimated parameters. Indirect effects are estimated as products of the path between the predictor (i.e., perceived supervisor support or belief in a just world) and the mediator (i.e., bullying) and the path between the mediator and the outcome (i.e., emotional exhaustion). The bootstrap analyses revealed that the indirect effects of perceived supervisor support (β = −0.22, CI = [–0.28, −0.17], *p* < 0.01) and belief in a just world (β = −0.09, CI = [–0.13, −0.06], *p* < 0.01) on emotional exhaustion through bullying were significant.

## 4. Discussion

Often, psychosocial risks are studied separately, even though the literature shows destructive effects of cumulated risks [29]. We have tried here to understand the common origins of two major psychosocial and mental factors that are harmful, namely, workplace bullying and emotional exhaustion. Our study was aimed at gaining a better understanding of the relationships between bullying at work, burnout, and their common causes in terms of lack of supervisor support and low belief in a just world. The rationale behind our focus on the relationships between burnout, bullying, and social support from one’s supervisor was twofold. First, social support is known to be a psychosocial predictor of both burnout and bullying. Second, the relationships between burnout and bullying need to be clarified due to the lack of scientific studies that look simultaneously at these two concepts. Moreover, these relationships need to be explored in relation to supervisor support and BJW.

All of the findings reported here suggest that social support at work is a crucial and independent aspect of the psychosocial environment influencing psychological health on the job. Confirming Hypothesis 1, our data showed that the lack of social support from one’s supervisor directly contributes to increasing emotional exhaustion. According to some scholars [5,44], a lack of support can trigger bullying directly, but it can also contribute to creating a stressful work climate in which bullying can flourish. In fact, the effect of such a lack of support could lie in the victim’s emotions, as such shame and guilty responses can result in a self-incrimination phenomenon [76], which could reinforce the feeling of this lack of support. In addition, these emotions could induce withdrawal behaviors and victim silence. This could be why targets do not ask for help or social support from their superiors even in critical situations [77]. Conversely, supervisor support can act as a coping resource and is positively related to problem-focused coping strategies and negatively related to emotion-focused coping strategies [78]. In another study on responses to serious bullying attacks, bystanders (coworkers and supervisors) gave more support to victims [79].

Additionally, confirming Hypothesis 2, that perceived supervisor support is negatively related to workplace bullying, our results on supervisor support from above are in line with previous investigations [8]. As has been found elsewhere [43], supportive and constructive leadership is associated with lower levels of bullying. In the face of situations of psychological stress, supervisors and coworkers appear to be the most important source of support [80], particularly when organizational support is lacking. In fact, supervisor support has many positive effects. For example, personal support given from one’s supervisor is strongly and positively related to nurses’ and nurse aides’ affective commitment [81].

Isolation, mistreatment, verbal abuses, and withdrawal from healthy relations generate negative effects related to feelings of distress and guilt. Negative attacks, humiliation, and associated “parasitic” affects (guilt, shame) induce extreme exhaustion. Confirming Hypothesis 3, our data revealed that exposure to workplace bullying is positively related to emotional exhaustion. In line with previous studies indicating links between workplace bullying and burnout [38,48,49,51,53], the present study confirms that workplace bullying can contribute to burnout. Our findings have several implications. In order to avoid confusion and to better understand the relationships between burnout and bullying, it is important to control, when studying burnout, the proportion of bullied workers. As numerous studies have shown, bullied workers have significantly higher levels of emotional exhaustion compared with non-bullied colleagues.

According to the conservation of resources theory [15], bullying is a potential mechanism via which employees’ resources are depleted, predisposing them to high levels of burnout. COR theory proposes that the workplace demands that individuals face require them to tap into available resources. For the COR theorists, bullying can be considered as a dramatic and dangerous loss of resources. As a consequence, the main and dramatic effect of the loss of resources affects health and particularly exhausts employees.

Moreover, our results confirm Hypothesis 4, that the lack of support from hierarchy is conductive to the development of workplace bullying that in turn promotes emotional exhaustion. Exposure to bullying behaviors is associated with an increase in psychological health problems, psychosomatic complaints, and negative state affectivity [11]. The present study is unique because it explored the potential mediating mechanisms of bullying between BJW, supervisor support, and burnout. The results confirmed the partially mediating role of bullying and provides insight into how supervisor support and BJW function as protective resources.

Our results demonstrate that the personal BJW of a bullied employee can contribute in distinct ways to reducing emotional exhaustion. Our results confirmed Hypothesis 5, that BJW and emotional exhaustion are directly related. However, in validation of Hypothesis 6, this route is indirect, because BJW can be affected by bullying, which in turn can increase exhaustion. Studies [68] have shown that the positive function of BJW may be reduced because repeated and prolonged exposure to mobbing has adverse effects on BJW. Stated differently and in line with our model, individuals who believe that what happens to them is fair and that they deserve what they get probably feel less exhausted because their perception of being bullied is mitigated. Our study showed, lastly, that BJW is an important belief that plays a strong role in protecting employees from bullying and exhaustion. Our results confirm the adaptive functions of BJW [14]. According to the prevention view [18], BJW directly and indirectly helps individuals reduce negative psychological symptoms, particularly emotional exhaustion, as shown in [19]. Moreover, BJW helps individuals interpret uncertain, uncontrollable, or difficult work factors in meaningful ways. Additionally, BJW is protective because it sustains trust in other employees and enhances a person’s trust that he/she will be treated fairly by others (supervisor and coworkers). According to the promotion view, in which individual differences in BJW are positively related to more general levels of well-being and/or positive feelings about oneself [20], BJW helps individuals maintain positive emotions and psychological states. More generally, as supported by [59,60], BJW provides a general framework for interpreting events and experiences in one’s own life and is thus an important and widely applicable personal resource.

Another paradox and confounding factor inherent in this kind of study is that very often the bullier is a supervisor [41,82]. Clearly, it is impossible to ask for and receive help from those who themselves are aggressors and have full power. Recognition of the need for help from other managers is necessary in these problematic situations. Additionally, a BJW may precisely be a second essential resource. Our results have shown that, apart from the social support hierarchy, reducing the level of emotional exhaustion, requires strengthening one’s BJW, which then becomes an interesting psychosocial lever against bullying acts. An organization that develops in-house fairness may contribute to enhancing the belief in a just world [65].

### Study Limitations

As in all research, this study has limitations. First, the present cross-sectional study does not allow us to draw any conclusions about causal relations or changes over time in the predictors of bullying and burnout. Longitudinal or experimental study designs are needed to test the causal effects of support on both bullying and burnout. Additionally, longitudinal designs would be useful for gaining further insight into the relationships hypothesized to exist in this study. Secondly, self-reported questionnaires can favor socially desirable responses. Added to that, all of our information was obtained from the same source. Those two circumstances could cause problems related to the common variance [83]. For future investigations, it might be interesting to obtain objective information about the sources and kinds of social support from supervisors, to distinguish between different kinds of leadership (transitional, transformational, empowerment, etc.), the number of agents assigned to the team, the worker/supervisor ratio, etc. A third limitation is linked to our participants. The sample was large and allowed us to generalize our findings to many jobs, but it was difficult to draw conclusions because different jobs have different work conditions. Indeed, the individuals sampled in this study were relatively heterogeneous, including workers from diverse companies (public and private) and from the tertiary and secondary sectors. Another limitation is the lack of data on environmental factors (company climate, size, etc.) in our sample, although research suggests that these added factors may contribute to emotional exhaustion. A final limitation is related to our mediations. In the light of studies measuring the mediating roles of need satisfaction [53], it is necessary to measure need-thwarting as a mediator in the links we have explored.

## 5. Practical Implications and Interventions

At the primary prevention level, it is necessary to report risk factors and devise campaigns. Additionally, managers can foster a cooperative and helpful climate, promote organizational citizenship, and banish competitive management. Having anti-bullying policies that recognize the role of managers and bystanders could be an important step. Education about bystanders’ responsibilities at work and prevention roles must be provided. The literature suggests that, in addition to training programs, managers should establish appropriate organizational and occupational values to stop employee habituation to an aggressive climate [82]. A zero tolerance approach, with a respectful work environment, is strongly advised.

Based on our findings, we can contend that promoting social support from colleagues and from the hierarchy is an important step in decreasing workplace bullying and emotional exhaustion. It is crucial for organizations to make every effort to find ways of increasing opportunities to obtain social support for all staff members. One of the most effective remedies is social support from one’s supervisor. Supervisors play a very important protective role by valorizing recognition and gratitude, highlighting the capacities and abilities of subordinates and helping them understand their work and tasks via direct assistance (instrumental or psychological). Encouraging social support in an organization is critical. All of the findings reported here suggest that social support at work is an essential, independent aspect of the psychosocial environment that affects psychological health on the job (bullying and burnout). Supportive managerial behaviors, such as understanding and acknowledging the employee’s perspective, providing meaningful information, offering opportunities for making choices and decisions, and encouraging initiative-taking [84], will reduce exhaustion and enhance well-being. If managers are engaged in positive supporting behaviors [85,86] and are encouraged and trained to act in a supportive manner, subordinates will display better physical and psychological well-being. In line with other results [87], our study lends credence to the idea that highly humane cultures emphasize the need for leaders or managers to be supportive and caring in their relationships with subordinates. Such cultures are likely to disapprove of bullying. More specifically, an interesting study [88] examined the extent to which leader–member exchanges (LMXs) affect the use of five strategies for coping with actual exposure to workplace bullying. The findings indicated that LMX quality significantly influenced the strategic use of acquiescence, exit, and retribution. Thus, it is important for institutions to pay particular attention to finding ways of increasing opportunities to obtain social support for all staff members and all parties involved. Social support could be improved by creating more exchanges between supervisors and subordinates and reinforcing organizational support and the security climate [89,90]. Managers could be trained to better supervise their subordinates by recognizing them, learning how to achieve appropriate communication with them, offering positive and valorizing feedback, and protecting their subordinates from poor working conditions.

At the secondary prevention level, human resource management (HRM) has to ensure that deviance and antisocial or aggressive behaviors at work are not condoned and are strictly prohibited. HRM has to quickly react and be attentive to the work climate and to employees’ beliefs and feelings. Together with the employee, supervisors and counselors (occupational physicians, occupational nurses, labor psychologists, ergonomists, etc.) should attempt to identify and reduce interpersonal conflicts related to bullying.

At the same time, we encourage the development of personalized accompaniment (tertiary prevention), which helps employees understand the positive or negative roles played by their beliefs and interpretations of their work environment as just or unjust. Thinking (or even worse, accepting) that one’s work environment is unfair cannot help workers and is a vector via which bullying can have destructive effects like exhaustion. Conversely, thinking that the work environment is just, being indignant about unfairness or bullying, and continuing to trust the company can help maintain balance and generate a positive gain spiral: social support (from supervisors). Augmented job resources (efficacy and just-world beliefs) can lead to decreases in poor job attitudes and behaviors (conflicts, feeling of isolation, lack of motivation, loss of meaningfulness, etc.), which in turn can further increase work engagement. Proactive interventions of specialists must help victims develop coping skills and become more resilient for handling such situations and quickly reacting and consulting qualified personnel. In order to come out of the process of bullying without leaving the company, it is necessary [82] to insist on the seriousness of all acts (and of isolation). The bullied person should not be introduced as a stigmatized person but as a prosocial employee able to forsake any negative information. At the same time, specific treatment for depressive symptoms must be proposed and provided to victims by qualified personnel.

## 6. Conclusions

Our objective was to examine the role of both supervisor support and belief in a just world on exhaustion in critical situations involving bullying. The idea that the relationship between supervisor support and burnout might be mediated by bullying proved to be a fruitful approach, and exploring social support from one’s supervisor can help us better understand suffering at work and potential routes toward remediation. Based on our findings, we can contend that both promoting supervisor support and using beliefs as resources are needed to decrease workplace bullying and emotional exhaustion.

## Figures and Tables

**Table 1:** Correlation matrix for the study variables.

Variables	*M*	*SD*	1	2	3	4
1. Supervisor support	2.76/4	0.65	**0.78**			
2. Belief in a just world	3.07/5	0.96	0.42	**0.78**		
3. Bullying	1.39/5	0.44	–0.56	–0.40	**0.93**	
4. Emotional exhaustion	3.23/7	1.64	–0.43	–0.42	0.59	**0.89**

Note: All correlations are significant (*p* < 0.001). Cronbach alpha are in bold in the diagonal.
